# HLA A*32 is associated to HIV acquisition while B*44 and B*53 are associated with protection against HIV acquisition in perinatally exposed infants

**DOI:** 10.1186/s12887-019-1620-6

**Published:** 2019-07-23

**Authors:** Linda Mouafo Mekue, Céline Nguefeu Nkenfou, Elvis Ndukong, Leaticia Yatchou, Beatrice Dambaya, Marie-Nicole Ngoufack, Joel Kadji Kameni, Jules-roger Kuiaté, Alexis Ndjolo

**Affiliations:** 1 Faculty of Science, University of Dschang, P.O. Box 56, Dschang, Cameroon; 2 Chantal BIYA International Reference Centre, P.O. Box 3077, Yaounde, Cameroon; 3 Laboratory of Animal Physiology and Health, Institute of Agriculture Research for Development (IRAD), P.O Box 2123, Bambui, Cameroon; 4 Higher Teacher Training College, University of Yaounde I, P.O. Box 47, Yaounde, Cameroon; 5 Faculty of Medicine and Biomedical Science, University of Yaounde I, P.O BOX 1364, Yaounde, Cameroon

**Keywords:** HIV, Mother-to-child transmission, HIV acquisition, HLA genotype, Haplotype, Homozygosity, Cameroon

## Abstract

**Background:**

Human leukocyte antigen (HLA) molecules play a key role in the cellular immune system. They may be determinants of mother-to-child transmission which is the driving force in pediatric HIV infection. We intended to look at the impact of the distribution of these polymorphic HLA genes in the mother-to-child transmission (MTCT) of HIV in Cameroon.

**Methods:**

A total of 156 mother-baby pairs were enrolled in three hospitals of Yaounde, capital of Cameroon. After the extraction of the DNA from blood samples using the Qiagen Kit as per manufacturer’ instructions, the polymorphism of the *HLA* class 1 ABC was determined using the PCR- sequence specific primers assay.

**Results:**

The distribution of *HLA* class 1 revealed that none of the allele studied was associated with transmitters or non-transmitters, so was not implicated in transmission. The regression analysis showed that HLA A*32 [OR 0.062 (CI; 0.0075 to 0.51)] is associated with HIV acquisition while HLA B*44 [OR 0.47 (CI; 0.21 to 1.14)] and HLA B*53 [OR; 0.14 (CI; 0.018 to 1.22)] were implicated in reducing the acquisition of HIV by infants. The homozygosity of locus C [OR 6.99 (CI; 1.81 to 26.88), *p* = 0.0027] was found as a risk factor for the acquisition, while the A*32-B*44 haplotype [OR 10.1 (CI 1.17 to 87.87), *p* = 0.03] was a risk factor for the transmission.

**Conclusion:**

This study has found that HLA A*32, B*44 and B*53 have an impact in MTCT outcomes. The homozygosity of locus C and the A*32-B*44 haplotype were risk factors for acquisition and transmission respectively.

**Electronic supplementary material:**

The online version of this article (10.1186/s12887-019-1620-6) contains supplementary material, which is available to authorized users.

## Background

More than three decades after the discovery of HIV-1, the number of infected children born to HIV infected mothers is still high. In Cameroon it was estimated at 4000 [1600–6500] in 2016 [1]. Despite prevention protocol there exist a residual vertical transmission of 2% [2]. Prevention of mother to child transmission of HIV started in 2000 and option B+ was adopted in 2012. Free ARV programme started in 2005 during which qualified patients were freely given ARV. Access to ARV treatment is free once presented at any treatment centre. The mother-to-child transmission (MTCT), the driving force of the pediatric infection can occurs in utero*, intrapartum and postpartum.* During these phases the immune system plays a role in reducing the viral load. High maternal viral load levels (mainly in the last months of pregnancy and/or during labour) increase the MTCT [3]. A part from viral load, other factors like genetic factors which are important players in the response to pathogens can also modulate the transmission. Because of its high rate of mutation HIV virus evade the immune response. So, understanding the implication of polymorphic HLA genes can provide a solution on how to manage HIV infection. Major histocompatibility complex (MHC) gene called Human leukocyte antigen (HLA) in human plays the role of antigen presentation between the immune system response and the virus. This is the most polymorphic gene in human [4], and this polymorphism result in variability in antigen presentation to host lymphocytes.

In fact many studies have identified some HLA alleles playing a role in the HIV sexual acquisition [5], infection progression to AIDS [6, 7] and vertical transmission [8, 9]. These factors may thus explain the 2% residual transmission occurring under preventive ART treatment or the 64% non-transmission without any prevention.

The aim of this study was to analyse the association between HLA genotypes, maternal homozygosity, mother-child allele concordance, haplotype and MTCT.

## Methods

### Definition of terms

Acquisition: The term acquisition is used when the factor is identified in babies.

Transmission: The term transmission is used when the factor is identified in mother.

### Study population

A total of 156 mother-baby couples were recruited for this study. Samples were collected from January 2014 to December 2016 in three hospitals of Yaounde. These mother-baby pairs were grouped as: 42 couples of transmitters (HIV infected mother, HIV infected baby), 64 couples of non-transmitters (HIV infected mother, non-infected baby) and 50 unrelated and healthy couples (HIV non infected mother, HIV non infected baby) as controls. Enrolled mothers were aged between 17 and 46 years and their babies from 1 to 59 weeks old. A total of 93% delivered by vaginal route. Treatment duration varied from 3 months to 5 years. Those HIV infected mothers not on treatment at the time of study were referred to treatment centre for ARV initiation.

### HLA genotyping

Genomic DNA was extracted from buffy coat using Qiagen QIAamp DNA blood kit according to the manufacturer’ instructions.

HLA typing, of class I HLA-A, HLA-B, and HLA-C loci, was performed by standard sequence specific primer polymerase chain reaction (SSP-PCR) using kits from One Lambda company (One Lambda, 21001 Kittridge St. Canoga Park, CA 91303–2801, USA). Briefly, 5.6 μl of 0.1 U/μl AmpliTaq DNA polymerase were introduced in an eppendorf tube containing the PCR cocktail (contains 1.5 mM MgCl2, 50 mM KCl, 10 mM Tris-HCl, 0.001% gelatine, glycerol and cresol red). This mixture was then vortexed and the well of the negative control filled. One hundred eleven μl of DNA was added to the rest of the mixture. The tube containing this master mix was then vortexed and 10 μl pipetted into the rest of the wells in the PCR plate. The plate was then sealed properly with the PCR cover plate and transferred into the thermal cycler. Amplification was carried out under conditions set by the manufacturer. Amplicons obtained were then submitted to a 2% agarose gel electrophoresis. After migration, the code of wells containing the positive amplifications were noted. These codes and the picture of the gel were then introduced into the HLA Fusion 4.1 software (One Lambda 21001 Kittridge St Canoga Park, CA 91303–2801, USA), which analysed the gel’s bands and the different HLA class 1 alleles were obtained. A sample was confirmed when the software was able to give the two alleles A, two alleles B and two alleles C. The allele frequencies (AF) were also determined using Excel, version 2013.

### Mother-baby HLA concordance, maternal homozygosity, and haplotypes sorting

This was done as previously described by Mackelprang et al. in 2008 [9]. HLA concordance was scored as the number of shared class I alleles between mother and baby. Pairs were scored as having 2 matches at a locus if the mother was homozygous at that locus.

Maternal homozygosity was scored as any versus none, according to whether the mother was homozygous for at least 1 class I locus.

The different haplotypes analysed were chosen from the combination of alleles having an impact in the acquisition, or haplotypes that have been found in a previous studies from Cameroon, or from other countries having an impact on MTCT.

### Statistical analyses

The significance of difference in allele frequencies of HLA-A, −B, HLA-C, alleles between patients and controls, transmitters and non-transmitters were compared by Chi-square test with Bonferroni correction and Fisher’s exact test. Odds ratio (ORs) and 95% confidence intervals (CIs) were calculated to determine levels of significance. For all tests, a probability (p) of less than 0.05 was considered significant.

## Results

### Study population

The HLA genotyping was done from of a total of 156 mothers, each with her baby. They were made up of 42 transmitters, 64 non-transmitters and 50 HIV non-infected. In the transmitters group 45.2% (19/42) of mothers were under prophylaxis/treatment and 54.8% (23/42) without any prevention protocol. In the non-transmitters group 46.9% (30/64) of mothers were under prophylaxis/treatment and 53.1% (34/64) without any prevention protocol (Table 1).

### HLA class 1 ABC genotypes in the study population

**Fig. 1:**
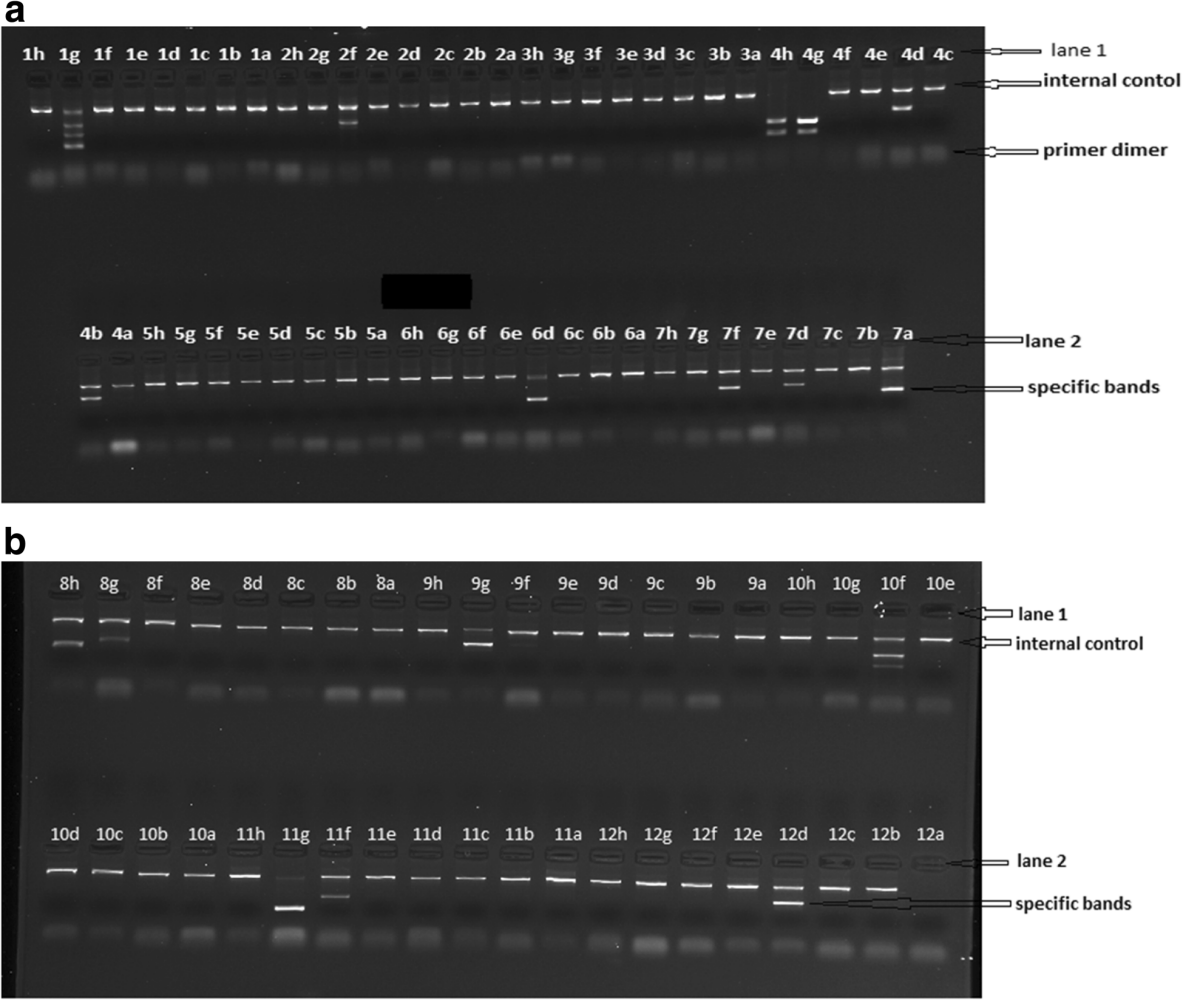
Electrophoregram of a maternal sample for the HLA ABC genotyping. *The specific expected bands are found between the internal control and the primer dimer. Positive reaction (gel (****a****): 1 g, 2f, 4 h, 4 g, 4d, 4b, 6d, 7f, 7d,7a; gel (****b****): 8 h, 8 g,9 h,10f,11 g,11f,12d), negative control (12a)*

The two images below (Fig. 1 (a) and (b)) illustrate the bands on the electrophoregrams obtained after the migration of a PCR amplification of one mother’ sample. One sample was ran on two agarose gels and the various positivities were recorded *(gel (a): 1 g, 2f, 4 h, 4 g, 4d, 4b, 6d, 7f, 7d,7a; gel (b): 8 h, 8 g,9 h,10f,11 g,11f,12d).* From these recorded positivities, the genotype sorted by the software (HLA fusion V 4.0) was: HLA-A*29, A*30, HLA-B*07, B*53, and HLA-C*02, C*07, because every human is supposed to have two alleles (locus) per gene (A, B and C)*.*

**Table 1:** Characteristics of the mothers enrolled in the study

	Age (Years for mothers, weeks for babies)	CD4 (cells/ml)	Viral load (Log_10_ RNA copies/ml)
Mothers			
T (*n* = 42)	27.1 ± 6.4	446.5 (62–880)	4.48 (ND-5.08)
NT (*n* = 64)	29.1 ± 4.4	448.5 (250–756)	3.8 (ND-4.94)
Controls *(n* = 50)	27.1 ± 6.5	/	/
Babies			
EI (*n* = 42)	21.1 ± 13.8	1724.5 (621–3050)	4.38 (2.5–8.1)
ENI (*n* = 30)	11.2 ± 7.9	/	/
healthy *(n* = 50)	25.9 ± 14.4	/	/

Continuous variables are presented as (Mean ± SD) and [median (range)]. ND: stands for Non Detectable

*T* transmitter, *NT* non-transmitter, *EI* exposed infected, *ENI* exposed non-infected; / non applicable

From our study population we have 71 alleles of HLA: *HLA-B* locus showed the greatest allelic diversity with 32 alleles, followed by *HLA-A* with 25 alleles and *HLA-C* showed the least amount of diversity with 14 alleles observed (Additional file 1). The most represented alleles were HLA A*02 (AF: 26.4%), HLA B*07 (AF: 13.8%) and HLA C*07 (AF: 23.1%). Eight rare *HLA-A* and 13 *HLA-B* alleles were also observed (rare alleles refer to those with AF less than 1% in a study population). Overall, deviation from Hardy–Weinberg equilibrium was not observed in the distribution of all the HLA genotypes in this population (*P* > 0.05).

### Phenotype frequencies distribution between transmitters and non-transmitters: impact on mother-to-child transmission

The phenotype of each of the different allele, was obtained by counting the number of all the participants having that allele in their genome.

HLA B*44 (26.4% Vs. 12%, *P* = 0.04) and C*02 (26.4% Vs. 12%, *P* = 0.04) were found to be more frequent in the HIV infected mothers than in the non-infected, while B*13 (10% Vs. 0.9%, *P* = 0.006) was rather higher in the HIV non-infected mothers (Additional file 2). No significant difference in the distribution of these allele was found between the transmitters and non-transmitters (Additional file 3). When the transmitters and non-transmitters were subdivided into those with PMTCT and those without, we found that HLA B*44 (38.2% Vs 16.7%, *p* = 0.03), A*02 (66.7% Vs. 29.4%, *p* = 0.01) and HLA A*36 (0% Vs. 17.6%, *p* = 0.01) were higher in the non-transmitters without treatment, while HLA C*12 (20% Vs. 2.9%, *p* = 0.01) was higher in non-transmitters with treatment. HLA A*23 (0% Vs. 21.7%, *p* = 0.03) was higher in the transmitters without treatment/prevention protocol.

### Alleles distribution between HIV exposed and non-exposed non-infected babies: impact in the acquisition

As with the mothers, the frequencies of various phenotypes were calculated in babies. Concerning the difference between the groups of HIV exposed (infected and non-infected) and non-exposed, B*14 and B*44 were the only alleles showing a difference in distribution: 4.7% Vs. 16%, *P* = 0.01 and 35.8% Vs. 14%, *P* = 0.004 respectively (Additional file 4). Between exposed infected and exposed non-infected, the following alleles showed significant difference: A*32 (16.7% Vs. 1.6%, *P* = 0.005), B*44 (26.2% Vs. 42.2%, *P* = 0.04) and B*53 (2.4% Vs. 14.1%, *P* = 0.03) (Additional file 5), in other words A*32 was higher in the exposed infected, while B*44 and B*53 were higher in the exposed uninfected. The regression logistic analysis looking at the implication of these alleles gave the following odds ratio and *p* value: B*44 (OR 0.47; 95% CI 0.21–1.14, *P* = 0.04), B*53 (OR 0.1491; 95% CI 0.018–1.22, *P* = 0.03) and A*32 (OR 0.062; 95% CI 0.0075–0.51, *P* = 0.001). We could suggest from these findings that B*44, A*32 and B*53 are involved in the outcome of vertical HIV exposition: HLA B*44 and B*53 are associated to protection against HIV acquisition, meanwhile HLA A*32 is associated with HIV acquisition in vertically exposed children.

### Mother-baby allele concordance, homozygosity and haplotypes impact on the transmission

Table 2 below shows the level of allele concordance between the mothers and the babies. We found that sharing of alleles did not have a significant impact in the transmission, but we instead noticed that only among the transmitters up to 6 alleles were shared between mother and baby. The homozygosity impact in the transmission or acquisition was also analysed. Only homozygosity for the locus C [OR 6.99 (CI; 1.81 to 26.88), *p* = 0.0027] (Table 3), found highly in exposed infected babies, was a risk factor for the acquisition. The haplotype A*32-B*53 was completely absent from our study population (Table 4). A*02-C*16, A*02-B*45 and A*24-B*44 did not show any significant difference when comparing the transmitters and non-transmitters. The A*32-B*44 [OR 10.1 (CI 1.17 to 87.87), *p* = 0.03] expressed highly in the transmitters was found to be a risk factor for the transmission.

**Table 2:** Allele concordance and risk of transmission

Number of shared alleles	NT (*N* = 64) n (%)	T (*N* = 42) n (%)	OR (95%CI)	p
4	25 (40.3)	13 (30.9)	0.66 (0.28 to 1.52)	0.33
5	5 (8.1)	5 (11.9)	1.54 (0.41 to 5.69)	0.51
6	/	2 (4.8)	/	/

*T* transmitter, *NT* non-transmitter, *N* number of mother within the group, *n* number of mother expressing the observation

**Table 3:** Homozigosity distribution and implication in the transmission

HLA Locus	mothers		OR (95% CI)	*P* value	Babies		OR (95% CI)	*P* value
	NT n (%)	T n (%)			EI n (%)	ENI n (%)		
A	10 (16.1)	5 (11.9)	0.70 (0.22 to 2.22)	0.77	6 (14.3)	9 (14.5)	0.98 (0.32 to 2.99)	1
B	7 (11.3)	5 (11.9)	1.07 (0.31 to 3.60)	0.92	1 (2.4)	2 (3.2)	0.73 (0.06 to 8.34)	0.80
C	6 (9.7)	6 (14.3)	1.55 (0.46 to 5.19)	0.53	11 (26.2)	3 (4.8)	6.99 (1.81 to 26.88)	0.0027^#^

^#^significantly different *p*-value

*n* number of mother expressing the observation, *T* transmitter, *NT* non-transmitter, *EI* exposed infected, *ENI* exposed non-infected

**Table 4:** Haplotype distribution and impact in the transmission

	T n (%)	NT n (%)	OR (95%CI)	*P* value
A*32-B*53	/	/	/	/
A*32-B*44	6 (11.9)	1 (1.6)	10.1 (1.17 to 87.87)	0.03^#^
A*02-C*16	2 (4.8)	3 (4.8)	0.98 (0.15 to 6.15)	0.98
A*02-B*45	1 (2.4)	3 (4.8)	0.47 (0.04 to 4.77)	0.53
A*24-B*44	1 (2.4)	1 (1.6)	0.90 (0.13 to 5.55)	0.66

*n* number of mother expressing the observation, *T* transmitter, *NT* non-transmitter

^#^significantly different *p*-value

## Discussion

The highlighted findings of this study are as follow: we had a highly polymorphic sample population, with 71 alleles of HLA: 25 HLA class A, 32 HLA class B and 14 HLA class C. HLA A*02, HLA B*07 and HLA C*07 were the most represented. HLA B*44 was more frequent in the HIV infected mothers, but within this group it was higher in the non-transmitters. Concerning the acquisition, B*44 was the only allele showing a differential distribution between HIV exposed infected babies and HIV exposed non infected babies. The allele A*32 was associated with acquisition meanwhile B*44 and B*53 were associated with protection. The homozigosity for the locus C in children was associated with the acquisition, and the haplotype A*32-B*44 was associated to the transmission.

Data obtained have revealed that most of the identified alleles were associated with HIV as in other studies. A review in 2001 by Trachtenberg and Erlich of published works has reported an HLA association with HIV transmission and disease progression to AIDS [10]. More studies showed these HLA associations [8, 9, 11]. In our study, HLA-A*02 appeared to be the most frequent allele as in other populations from Cameroon [12, 13]. HLA-A*02 was shown to stimulate peripheral blood mononuclear cells in a manner that inhibits HIV replication. This has been stated to be the reason for a documented 9-fold reduced risk of HIV transmission to infants during childbirth [14], but in contradiction we have not found it to be implicated in MTCT. Contradicting studies showing the implication of these alleles’ haplotypes in increasing the viral load were published. In fact HLA-A*02-C*16 and HLA-A*02-B*45 have been shown to contribute significantly in increasing viral loads (greater than 100,000 copies per milliliter) [6]. A recent study has found that vaccine efficacy in the RV144 HIV-1 vaccine trial was greater for participants who expressed HLA-A*02 [15].

HLA B*07, the most frequent in our study population, is a worldwide frequent HLA B allele. HLA-B*07:02 was associated with disease progression in a B-clade from Europe [7], but this was not the case in a large cohort of 1,210 C-clade-infected individuals from Durban [16]. This allele tends to be in linkage disequilibrium with the HLA C*07 in the HIV disease-susceptible implication [7]. The HLA C*07 is one of the highly frequent HLA-C allele and the most common, divergent, and polymorphic [17]. Allotype of this allele has been found to be associated with either a low (HLA-C*07:01) or an increased (HLA-C*07:02) rate of seroconversion in a Kenyan population [5].

We showed elsewhere that a certain cluster of functionally related class I MHC allele, HLA B*44, was associated with reduced risk of acquisition of HIV by infants in a population of Cameroon [13]. We sought to determine whether this same HLA is associated with protection from HIV-1 MTCT in an independent population. This protective role was confirmed in the present study population. Nevertheless its frequency was higher in infected mothers than the non-infected. We may think that this allele protects from acquisition and MTCT, but not from the sexual or other mode of transmission. Reports of HIV-1 clearance in perinatally exposed infants and the presence of cytotoxic T cell responses against HIV-1 in exposed uninfected infants suggests that, in some circumstances, fetuses or new-borns can abort or eliminate infection through virus-specific cellular effector mechanisms [18]. One important determinant of resistance and susceptibility to infection is the MHC, the class I alleles determine the molecular targets of the cytotoxic T lymphocytes (CTL) in a given host.

Studies looking at the implication of HLA B*53 allele in HIV/AIDS infection are rare. A study from Gao et al. (2001) [19] found that most of the black people (African American) compared to whites carry this allele, and it leads to rapid progression. A recent study has concluded that HLA-B*53:01 molecules may be directly or indirectly involved in the mechanisms that induce drug reaction with eosinophilia and systemic symptoms (DRESS) syndrome in patients treated with raltegravir [20]. Concerning its implication in the MTCT of HIV, it has been found that mother bearing the B*53:01 did not transmit the virus despite high viral load [21], this is consistent with our findings. The contradiction between our results with those of Gao et al. may be explained by the fact that they were looking at the mechanisms of action of this allele in regards to an amino change, mainly at the molecular level. In fact they suggest that the difference in affinity for tyrosine at the carboxy-terminal position of the peptide may influence the relative efficiency of HLA-B*53:01 in presenting specific HIV-1 epitopes to cytotoxic T lymphocytes and may thereby account for different effects on progression to AIDS [19].

This study presents evidence of a significant association between HLA-A*32 and baby acquisition of HIV infection, contradicting other studies. This allele has been shown to be associated with slow disease progression in two related mixed population cohort studies [22, 23]. A study on HLA association with CTL response to novel HIV-1 vaccines showed favorable prognosis with A*32 [24]. In addition, a small transfusion study in a group of HIV-1 infected, long-term non progressor (LTNP) Australian Caucasians also showed a trend toward protection with A*32 [25]. This allele was also found to act in an haplotype form (A*32; Cw1; B*27; DR12 (DR5) in preventing the ankylosing spondylitis in Sardinian population [26]. These contradictions can be due to the differences of study populations.

We didn’t find any significant difference in regards to the concordance of allele between mother and baby. But it was found that those who shared more than five alleles were transmitters. This is in agreement with some previous results in a population from America [27] and a population from Kenya [9]. In fact the diversity of the HLA alleles, both class I and class II, broadens the repertoire of presentation and generates a greater diversity in the immune response. It has been shown that fetal cord blood leukocytes can expressed a strong response against foreign maternal MHC when different from his own [28]. The first exposure to HIV-1 in utero and during birth may involve free or, perhaps more likely, cell-associated virus. When challenged with HIV-1–infected maternal cells, the baby has the potential to respond to a different maternal HLA antigens. Haplotypes studied did not showed a significant impact on the transmission. Except the A*32-B*44 haplotype that was identified as favoring the transmission. This can be explained also by the fact that when there is concordance of allele in mother and baby, the immune system of the baby may not expressed an immune response against the foreign cells, hence favoring the transmission.

However, one of the limits of this study was the small size population, that can impact the power of the results. It is important to call upon the attention that Cameroon has a great ethnic diversity. Enrolment was done in Yaoundé, a cosmopolitan capital. It will be interesting to investigate the validity of these results in specific ethnic population.

## Conclusion

From our analyses, maternal HLA alleles previously associated with vertical HIV-1 transmission have different role than those of babies. In babies, different HLA alleles are associated with acquisition of infection. Likewise, the three HLA class I alleles in babies (A*32, B*44 and B*53) implicated in HIV-1 acquisition clearly lack the protective role when present in mothers. Therefore, within paired mother-baby, there is more to be learned about the mechanisms of adaptive and innate immunity that control the process of viral transmission as distinct from those that mediate viral acquisition.

## Additional files

Additional file 1 : **Table S1.** Frequencies of HLA-A, HLA-B and HLA-C alleles in the study population. (DOCX 29 kb)

Additional file 2 : **Table S2.** HLA class 1 ABC distribution in the HIV infected and non-infected mothers. (DOCX 38 kb)

Additional file 3 : **Table S3.** HLA class 1 ABC distribution in the non-transmitters and transmitters. (DOCX 36 kb)

Additional file 4 : **Table S4.** HLA class 1 ABC distribution in HIV exposed and non-exposed babies. (DOCX 36 kb)

Additional file 5 : **Table S5.** HLA class 1 ABC distribution in the HIV exposed infected and exposed non-infected babies. (DOCX 34 kb)

## Data Availability

Provided upon request.

## Abbreviations

AF: Allele frequency
ARV: Anti-retroviral
CI: Confidence Interval
CTL: Cyto toxic Lymphocyte
EI: Exposed Infected
ENI: Exposed Non Infected
HIV: Human Immunodeficiency Virus
HLA: Human Leucocyte Antigen
MTCT: Mother-to-child transmission
NT: Non transmitter
OR: Odds Ratio
T: Transmitter
