# Supplementary material for: HLA A*32 is associated to HIV acquisition while B*44 and B*53 are associated with protection against HIV acquisition in perinatally exposed infants
Source: BMC Pediatr. 2019 Jul 23;19:249. doi: 10.1186/s12887-019-1620-6 (PMC6647251; doi:10.1186/s12887-019-1620-6)
Supplement: Supplementary file 1 — : Table S1. Frequencies of HLA-A, HLA-B and HLA-C alleles in the study population. (DOCX 29 kb) [file 12887_2019_1620_MOESM1_ESM.docx]

**Additional file 1: Table S1:** Frequencies of HLA-A, HLA-B and HLA-C alleles in the study population

| **HLA class A** | **Allelic frequency [N (%)]** | **HLA class B** | **Allelic frequency [N (%)]** | **HLA class C** | **Allelic frequency [N (%)]** |
| --- | --- | --- | --- | --- | --- |
| **A*01** | 38 (6.1) | **B*07** | 81 (13.0) | **C*01** | 8(1.3) |
| **A*02** | 165(26.4) | **B*08** | 13(2.1) | **C*02** | 75(12.0) |
| **A*03** | 34(5.4) | **B*13** | 9(1.4) | **C*03** | 34(5.4) |
| **A*06** | (0.3) | **B*14** | 30(4.8) | **C*04** | 83(13.3) |
| **A*07** | 2(0.3) | **B*15** | 44(7.1) | **c*05** | 8(1.3) |
| **A*08** | 1(0.2) | **B*18** | 20(3.2) | **C*06** | 91(14.6) |
| **A*11** | 10(1.6) | **B*27** | 12(1.9) | **C*07** | 144(23.1) |
| **A*15** | 1(0.2) | **B*32** | 1(0.2) | **C*08** | 43(6.9) |
| **A*21** | 1(0.2) | **B*33** | 1(0.2) | **C*12** | 27(4.3) |
| **A*23** | 44(7.1) | **B*35** | 68(10.9) | **C*14** | 29(4.6) |
| **A*24** | 7(1.1) | **B*37** | 8(1.3) | **C*15** | 17(2.7) |
| **A*25** | 4(0.6) | **B*38** | 6(1.0) | **C*16** | 20(3.2) |
| **A*26** | 8(1.3) | **B*39** | 5(0.8) | **C*17** | 34(5.4) |
| **A*28** | 2(0.3) | **B*40** | 19(3.0) | **C*18** | 11(1.8) |
| **A*29** | 38(6.1) | **B*41** | 4(0.6) |  |  |
| **A*30** | 88(14.1) | **B*42** | 25(4.0) |  |  |
| **A*31** | 27(4.3) | **B*44** | 77(12.3) |  |  |
| **A*32** | 25(4.0) | **B*45** | 17(2.7) |  |  |
| **A*33** | 27(4.3) | **B*46** | 1(0.2) |  |  |
| **A*34** | 9(1.4) | **B*47** | 12(1.9) |  |  |
| **A*36** | 15(2.4) | **B*48** | 6(1.0) |  |  |
| **A*66** | 21(3.4) | **B*49** | 20(3.2) |  |  |
| **A*68** | 34(5.4) | **B*50** | 6(1.0) |  |  |
| **A*74** | 15(2.4) | **B*51** | 22(3.5) |  |  |
| **A*80** | 6(1.0) | **B*52** | 2(0.3) |  |  |
|  |  | **B*53** | 37(5.9) |  |  |
|  |  | **B*56** | 2(0.3) |  |  |
|  |  | **B*57** | 7(1.1) |  |  |
|  |  | **B*58** | 63 (10.1) |  |  |
|  |  | **B*73** | 1(0.2) |  |  |
|  |  | **B*81** | 4(0.6) |  |  |
|  |  | **B*82** | 1(0.2) |  |  |

*N: number of allele*
