# Supplementary material for: HLA A*32 is associated to HIV acquisition while B*44 and B*53 are associated with protection against HIV acquisition in perinatally exposed infants
Source: BMC Pediatr. 2019 Jul 23;19:249. doi: 10.1186/s12887-019-1620-6 (PMC6647251; doi:10.1186/s12887-019-1620-6)
Supplement: Supplementary file 2 — : Table S2. HLA class 1 ABC distribution in the HIV infected and non-infected mothers. (DOCX 38 kb) [file 12887_2019_1620_MOESM2_ESM.docx]

**Additional file 2: Table S2**: HLA class 1 ABC distribution in the HIV infected and non-infected mothers

| \| **HLA class A** \| **Phenotypic frequency**  **[N (%)]** \| \| ***P-value** \| **HLA class B** \| **Phenotypic frequency**  **[N (%)]** \| \| ***P-value** \| **HLA class C** \| **Phenotypic frequency**  **[N (%)]** \| \| \| ***P-value** \| \| --- \| --- \| --- \| --- \| --- \| --- \| --- \| --- \| --- \| --- \| --- \| --- \| --- \| \|  \| **Non- infected mothers** \| **Infected mothers** \|  \|  \| **Non-infected Mothers** \| **Infected mothers** \|  \|  \| **Non-infected mothers** \| \| **Infected mothers** \|  \| \| **A*01** \| 8 (16) \| 11 (10.4) \| 0.31 \| **B*07** \| 14 (28) \| 21 (19.8) \| 0.25 \| **C*01** \| 2 (4) \| 2 (1.9) \| \| 0.43 \| \| **A*02** \| 20 (40) \| 48 (45.3) \| 0.53 \| **B*08** \| 2 (4) \| 4 (3.8) \| 0.94 \| **C*02** \| **6 (12)** \| **28 (26.4)** \| \| **0.04** \| \| **A*03** \| 6 (12) \| 11 (10.4) \| 0.76 \| **B*13** \| **5 (10)** \| **1 (0.9)** \| **0.006** \| **C*03** \| 5 (10) \| 12 (11.3) \| \| 0.81 \| \| **A*06** \| / \| 1 (0.9) \| / \| **B*14** \| 8 (16) \| 9 (8.5) \| 0.16 \| **C*04** \| 11 (22) \| 19 (17.9) \| \| 0.54 \| \| **A*07** \| 1 (2) \| / \| / \| **B*15** \| 9 (18) \| 13 (12.3) \| 0.33 \| **C*05** \| 1 (2) \| 3 (2.8) \| \| 0.75 \| \| **A*08** \| 1 (2) \| / \| / \| **B*18** \| 1 (2) \| 9 (8.5) \| 0.12 \| **C*06** \| 14 (28) \| 30 (28.3) \| \| 0.96 \| \| **A*11** \| 2 (4) \| 4 (3.8) \| 0.58 \| **B*27** \| 1 (2) \| 4 (3.8) \| 0.55 \| **C*07** \| 26 (52) \| 38 (35.8) \| \| 0.05 \| \| **A*15** \| / \| 1 (0.9) \| / \| **B*32** \| 1 (2) \| / \| / \| **C*08** \| 11 (22) \| 12 (11.3) \| \| 0.07 \| \| **A*23** \| 10 (20) \| 13 (12.3) \| 0.2 \| **B*35** \| 8 (16) \| 25 (23.6) \| 0.27 \| **C*12** \| 4 (8) \| 10 (9.4) \| \| 0.77 \| \| **A*24** \| / \| 1 (0.9) \| / \| **B*37** \| 1 (2) \| 4 (3.8) \| 0.55 \| **C*14** \| 2 (4) \| 13 (12.3) \| \| 0.11 \| \| **A*25** \| / \| 3 (2.8) \| / \| **B*38** \| / \| 3 (2.8) \| / \| **C*15** \| 6 (12) \| 5 (4.7) \| \| 0.09 \| \| **A*26** \| 1 (2) \| 1 (0.9) \| 0.94 \| **B*39** \| 1 (2) \| 2 (1.9) \| 0.96 \| **C*16** \| 1 (2) \| 9 (8.5) \| \| 0.12 \| \| **A*29** \| 4 (8) \| 12 (11.3) \| 0.52 \| **B*40** \| 2 (4) \| 8 (7.5) \| 0.40 \| **C*17** \| 9 (18) \| 9 (8.5) \| \| 0.08 \| \| **A*30** \| 15 (30) \| 24 (22.6) \| 0.32 \| **B*41** \| 2 (2) \| 1 (0.9) \| 0.5 \| **C*18** \| / \| 6 (5.7) \| \| / \| \| **A*31** \| 6 (12) \| 8 (7.5) \| 0.36 \| **B*42** \| 7 (14) \| 7 (6.6) \| 0.13 \|  \|  \|  \| \|  \| \| **A*32** \| 3 (6) \| 11 (10.4) \| 0.37 \| **B*44** \| **6 (12)** \| **28 (26.4)** \| **0.04** \|  \|  \|  \| \|  \| \| **A*33** \| 4 (8) \| 9 (8.5) \| 0.92 \| **B*45** \| 2 (4) \| 6 (5.7) \| 0.65 \|  \|  \|  \| \|  \| \| **A*34** \| 2 (4) \| 2 (1.9) \| 0.43 \| **B*47** \| 1 (2) \| 5 (4.7) \| 0.41 \|  \|  \|  \| \|  \| \| **A*36** \| / \| 7 (6.6) \| / \| **B*48** \| 1 (2) \| / \| / \|  \|  \|  \| \|  \| \| **A*66** \| 3 (6) \| 8 (7.5) \| 0.72 \| **B*49** \| 3 (6) \| 5 (4.7) \| 0.72 \|  \|  \|  \| \|  \| \| **A*68** \| 5 (10) \| 9 (8.5) \| 0.75 \| **B*50** \| 1 (2) \| 2 (1.9) \| 0.96 \|  \|  \|  \| \|  \| \| **A*74** \| 3 (6) \| (5.7) \| 0.93 \| **B*51** \| 2 (4) \| 9 (8,5) \| 0.30 \|  \|  \|  \| \|  \| \| **A*80** \| 1 (2) \| (3.8) \| 0.55 \| **B*52** \| / \| 1 (0,9) \| / \|  \|  \|  \| \|  \| \|  \|  \|  \|  \| **B*53** \| 7 (14) \| 11 (10,4) \| 0.50 \|  \|  \|  \| \|  \| \|  \|  \|  \|  \| **B*57** \| / \| 2 (1,9) \| / \|  \|  \|  \| \|  \| \|  \|  \|  \|  \| **B*58** \| 13 (26) \| 17 (16,0) \| 0.14 \|  \|  \|  \| \|  \| \|  \|  \|  \|  \| **B*73** \| / \| 1 (0,9) \| / \|  \|  \|  \| \|  \| \|  \|  \|  \|  \| **B*81** \| 1 (2) \| 2 (1,9) \| 0.96 \|  \|  \|  \| \|  \| \|  \|  \|  \|  \| **B*82** \| / \| 1(0,9) \| / \|  \|  \|  \| \|  \|   **P-value from chi-square/fisher test. Statistically significant results are marked in bold. /: Not applicable. N: number of phenotype* |  |  |  |  |  |  |  |  |
| --- | --- | --- | --- | --- | --- | --- | --- | --- | --- | --- | --- | --- | --- | --- | --- | --- | --- | --- | --- | --- | --- | --- | --- | --- | --- | --- | --- | --- | --- | --- | --- | --- | --- | --- | --- | --- | --- | --- | --- | --- | --- | --- | --- | --- | --- | --- | --- | --- | --- | --- | --- | --- | --- | --- | --- | --- | --- | --- | --- | --- | --- | --- | --- | --- | --- | --- | --- | --- | --- | --- | --- | --- | --- | --- | --- | --- | --- | --- | --- | --- | --- | --- | --- | --- | --- | --- | --- | --- | --- | --- | --- | --- | --- | --- | --- | --- | --- | --- | --- | --- | --- | --- | --- | --- | --- | --- | --- | --- | --- | --- | --- | --- | --- | --- | --- | --- | --- | --- | --- | --- | --- | --- | --- | --- | --- | --- | --- | --- | --- | --- | --- | --- | --- | --- | --- | --- | --- | --- | --- | --- | --- | --- | --- | --- | --- | --- | --- | --- | --- | --- | --- | --- | --- | --- | --- | --- | --- | --- | --- | --- | --- | --- | --- | --- | --- | --- | --- | --- | --- | --- | --- | --- | --- | --- | --- | --- | --- | --- | --- | --- | --- | --- | --- | --- | --- | --- | --- | --- | --- | --- | --- | --- | --- | --- | --- | --- | --- | --- | --- | --- | --- | --- | --- | --- | --- | --- | --- | --- | --- | --- | --- | --- | --- | --- | --- | --- | --- | --- | --- | --- | --- | --- | --- | --- | --- | --- | --- | --- | --- | --- | --- | --- | --- | --- | --- | --- | --- | --- | --- | --- | --- | --- | --- | --- | --- | --- | --- | --- | --- | --- | --- | --- | --- | --- | --- | --- | --- | --- | --- | --- | --- | --- | --- | --- | --- | --- | --- | --- | --- | --- | --- | --- | --- | --- | --- | --- | --- | --- | --- | --- | --- | --- | --- | --- | --- | --- | --- | --- | --- | --- | --- | --- | --- | --- | --- | --- | --- | --- | --- | --- | --- | --- | --- | --- | --- | --- | --- | --- | --- | --- | --- | --- | --- | --- | --- | --- | --- | --- | --- | --- | --- | --- | --- | --- | --- | --- | --- | --- | --- | --- | --- | --- | --- | --- | --- | --- | --- | --- | --- | --- | --- | --- | --- | --- | --- | --- | --- | --- | --- | --- | --- | --- | --- | --- | --- | --- | --- | --- | --- | --- | --- | --- | --- | --- | --- | --- | --- | --- | --- | --- | --- | --- | --- | --- | --- | --- | --- | --- | --- | --- | --- | --- | --- | --- | --- | --- | --- | --- | --- | --- | --- | --- | --- | --- | --- | --- | --- | --- | --- | --- | --- | --- | --- | --- | --- | --- | --- | --- | --- | --- | --- |
