# Supplementary material for: HLA A*32 is associated to HIV acquisition while B*44 and B*53 are associated with protection against HIV acquisition in perinatally exposed infants
Source: BMC Pediatr. 2019 Jul 23;19:249. doi: 10.1186/s12887-019-1620-6 (PMC6647251; doi:10.1186/s12887-019-1620-6)
Supplement: Supplementary file 3 — : Table S3. HLA class 1 ABC distribution in the non-transmitters and transmitters. (DOCX 36 kb) [file 12887_2019_1620_MOESM3_ESM.docx]

**Additional file 3: TableS3:** HLA class 1 ABC distribution in the non-transmitters and transmitters

| **HLA class A** | **Phenotypic frequency in** | | *** P-value** | **HLA class B** | **Phenotypic frequency in** | | ***P-value** | **HLA class C** | **Phenotypic frequency in** | | ***P-value** |
| --- | --- | --- | --- | --- | --- | --- | --- | --- | --- | --- | --- |
|  | **T [N (%)]** | **NT [N (%)]** |  |  | **T[N(%]** | **NT [N (%)]** |  |  | **T [N (%)]** | **NT [N (%)]** |  |
| **A*01** | 4(9.5) | 7 (10.9) | 0.25 | **B*07** | 8 (19.0) | 13 (20.3) | 0.19 | **C*01** | 1 (2.4) | 1 (1.6) | 0.48 |
| **A*02** | 18 42.9) | 30 (46.9) | 0.14 | **B*08** | 2 (4.8) | 2 (3.1) | 0.34 | **C*02** | 10 (23.8) | 18 (28.1) | 0.15 |
| **A*03** | 5 (11.9) | 6 (9.4) | 0.22 | **B*13** | 1 (2.4) | / | / | **C*03** | 5 (11.9) | 7 (10.9) | 0.24 |
| **A*06** | / | 1 (1.6) | / | **B*14** | 3 (7.1) | 6 (9.4) | 0.26 | **C*04** | 9 (21.4) | 10 (15.6) | 0.15 |
| **A*11** | 1 (2.4) | 3 (4.7) | 0.35 | **B*15** | 6 (14.3) | 7 (10.9) | 0.20 | **C*05** | / | 3 (4.7) | / |
| **A*15** | 1 (2.4) | / | / | **B*18** | 3 (7.1) | 6 (9.4) | 0.26 | **C*06** | 10 (23.8) | 20 (31.3) | 0.12 |
| **A*23** | 5 (11.9) | 8 (12.5) | 0.23 | **B*27** | 2 (4.8) | 2 (3.1) | 0.34 | **C*07** | 13 (31.0) | 25 (39.1) | 0.11 |
| **A*24** | / | 1 (1.6) | / | **B*35** | 12 (28.6) | 13 (20.3) | 0.11 | **C*08** | 5 (11.9) | 7 (10.9) | 0.24 |
| **A*25** | / | 3 (4.7) | / | **B*37** | 3 (7.1) | 1 (1.6) | 0.14 | **C*12** | 3 (7.1) | 7 (10.9) | 0.22 |
| **A*26** | / | 1 (1.6) | / | **B*38** | 2 (4.8) | 1 (1.6) | 0.28 | **C*14** | 6 (14.3) | 7 (10.9) | 0.20 |
| **A*29** | 7 (16.7) | 5 (7.8) | 0.09 | **B*39** | / | 2 (3.1) | / | **C*15** | 4 (9.5) | 1 (1.6) | 0.07 |
| **A*30** | 9 (21.4) | 15 (23.4) | 0.18 | **B*40** | 3 (7.1) | 5 (7.8) | 0.29 | **C*16** | 4 (9.5) | 5 (7.8) | 0.25 |
| **A*31** | 5 (11.9) | 3 (4.7) | 0.11 | **B*41** | / | 1 (1.6) | / | **C*17** | 3 (7.1) | 6 (9.4) | 0.26 |
| **A*32** | 7 (16.7) | 4 (6.3) | 0.06 | **B*42** | 2 (4.8) | 5 (7.8) | 0.26 | **C*18** | 3 (7.1) | 3 (4.7) | 0.28 |
| **A*33** | 3 (7.1) | 6 (9.4) | 0.26 | **B*44** | 10 (23.8) | 18 (28.1) | 0.15 |  |  |  |  |
| **A*34** | 1 (2.4) | 1 (1.6) | 0.48 | **B*45** | 2 (4.8) | 4 (6.3) | 0.32 |  |  |  |  |
| **A*36** | 2 (2.4) | 6 (9.4) | 0.12 | **B*47** | 2 (4.8) | 3 (4.7) | 0.35 |  |  |  |  |
| **A*66** | 3 (7.1) | 5 (7.8) | 0.29 | **B*49** | 2 (4.8) | 3 (4.7) | 0.35 |  |  |  |  |
| **A*68** | 5 (11.9) | 4 (6.3) | 0.16 | **B*50** | 1 (2.4) | 1 (1.6) | 0.48 |  |  |  |  |
| **A*74** | 2 (4.8) | 5 (6.3) | 0.32 | **B*51** | 4 (9.5) | 5 (7.8) | 0.21 |  |  |  |  |
| **A*80** | 2 (2.4) | 3 (4.7) | 0.35 | **B*52** | 1 (2.4) | / | / |  |  |  |  |
|  |  |  |  | **B*53** | 2 (4.8) | 9 (14.1) | 0.08 |  |  |  |  |
|  |  |  |  | **B*57** | 1 (2.4) | 1 (1.6) | 0.48 |  |  |  |  |
|  |  |  |  | **B*58** | 5 (11.9) | 12 (18.8) | 0.14 |  |  |  |  |
|  |  |  |  | **B*73** | / | 1 (1.6) | / |  |  |  |  |
|  |  |  |  | **B*81** | 1 (2.4) | 1 (1.6) | 0.48 |  |  |  |  |
|  |  |  |  | **B*82** | 1 (2.4) | / | / |  |  |  |  |

**P-value from chi-square/fisher test; T: transmitters; NT: non-transmitters; /: Not applicable. N: number of phenotype*
