# Supplementary material for: HLA A*32 is associated to HIV acquisition while B*44 and B*53 are associated with protection against HIV acquisition in perinatally exposed infants
Source: BMC Pediatr. 2019 Jul 23;19:249. doi: 10.1186/s12887-019-1620-6 (PMC6647251; doi:10.1186/s12887-019-1620-6)
Supplement: Supplementary file 4 — : Table S4. HLA class 1 ABC distribution in HIV exposed and non-exposed babies. (DOCX 36 kb) [file 12887_2019_1620_MOESM4_ESM.docx]

**Additional file 4: Table S4**: HLA class 1 ABC distribution in HIV exposed and non-exposed babies

| **HLA class A** | **Phenotypic frequency [N (%)]** | | ***P** | **HLA class B** | **Phenotypic frequency**  **[N (%)]** | | ***P** | **HLA class C** | **Phenotypic frequency**  **[N (%)]** | | ***P** |
| --- | --- | --- | --- | --- | --- | --- | --- | --- | --- | --- | --- |
|  | **HIV exposed** | **HIV non-exposed** |  |  | **HIV exposed** | **HIV non-exposed** |  |  | **HIV exposed** | **HIV non-exposed** |  |
| **A*01** | 14 (13.2) | 5 (10) | 0.56 | **B*07** | 24 (22.6) | 13 (26) | 0.64 | **C*01** | 2 (1.9) | 2 (4) | 0.43 |
| **A*02** | 51 (48.1) | 22 (44) | 0.63 | **B*08** | 3 (2.8) | 4 (8) | 0.14 | **C*02** | 28 (26.4) | 9 (18) | 0.24 |
| **A*03** | 13 (12.3) | 3 (6) | 0.21 | **B*13** | 2 (1.9) | 1 (2) | 0.96 | **C*03** | 10 (9.4) | 6 (12) | 0.62 |
| **A*06** | / | 1 (2) | / | **B*14** | **5 (4.7)** | **8 (16)** | **0.01** | **C*04** | 32 (30.2) | 14 (28) | 0.77 |
| **A*07** | / | 1 (2) | / | **B*15** | 14 (13.2) | 7 (14) | 0.89 | **C*05** | 3 (2.8) | 1 (2) | 0.75 |
| **A*11** | 3 (2.8) | 1 (2) | 0.75 | **B*18** | 6 (5.7) | 2 (4) | 0.65 | **C*06** | 29 (27.4) | 11 (22) | 0.47 |
| **A*21** | 1 (0.9) | / | / | **B*27** | 6 (5.7) | 1 (2) | 0.12 | **C*07** | 45 (42.5) | 26 (52) | 0.26 |
| **A*23** | 8 (7.5) | 9 (18) | 0.05 | **B*33** | / | 1 (2) | / | **C*08** | 10 (9.4) | 10 (20) | 0.06 |
| **A*24** | 5 (4.7) | 1 (2) | 0.41 | **B*35** | 24 (22.6) | 8 (16) | 0.33 | **C*12** | 10 (9.4) | 2 (4) | 0.23 |
| **A*25** | 1 (0.9) | / | / | **B*37** | 3 (2.8) | / | / | **C*14** | 10 (9.4) | 2 (4) | 0.23 |
| **A*26** | 6 (5.7) | / | / | **B*38** | 3 (2.8) | / | / | **C*15** | 2 (1.9) | 4 (8) | 0.06 |
| **A*28** | 2 (1.9) | / | / | **B*39** | 1 (0.9) | 1 (2) | 0.58 | **C*16** | 7 (6.6) | 3 (6) | 0.88 |
| **A*29** | 14 (13.2) | 5 (10) | 0.56 | **B*40** | 6 (5.7) | 3 (6) | 0.93 | **C*17** | 7 (6.6) | 8 (16) | 0.06 |
| **A*30** | 23 (21.7) | 14 (28) | 0.38 | **B*41** | 1 (0.9) | 1 (2) | 0.58 | **C*18** | 3 (2.8) | 2 (4) | 0.69 |
| **A*31** | 6 (5.7) | 7 (14) | 0.07 | **B*42** | 5 (4.7) | 6 (12) | 0.09 |  |  |  |  |
| **A*32** | 8 (7.5) | 3 (6) | 0.72 | **B*44** | **38 (35.8)** | **7 (14)** | **0.004** |  |  |  |  |
| **A*33** | 6 (5.7) | 6 (12) | 0.15 | **B*45** | 6 (5.7) | 3 (6) | 0.93 |  |  |  |  |
| **A*34** | 3 (2.8) | 2 (4) | 0.69 | **B*46** | 1 (0.9) | / | / |  |  |  |  |
| **A*36** | 8 (7.5) | / | / | **B*47** | 5 (5.7) | / | / |  |  |  |  |
| **A*66** | 6 (5.7) | 4 (8) | 0.57 | **B*48** | 3 (2.8) | 2 (4) | 0.69 |  |  |  |  |
| **A*68** | 10 (9.4) | 9 (18) | 0.12 | **B*49** | 7 (6.6) | 5 (10) | 0.45 |  |  |  |  |
| **A*74** | 4 (3.8) | 2 (4) | 0.94 | **B*50** | 2 (1.9) | 1 (2) | 0.96 |  |  |  |  |
| **A*80** | 1 (0.9) | / | / | **B*51** | 8 (7.5) | 3 (6) | 0.72 |  |  |  |  |
|  |  |  |  | **B*52** | 1 (0.9) | / | / |  |  |  |  |
|  |  |  |  | **B*53** | 10 (9.4) | 8 (16) | 0.23 |  |  |  |  |
|  |  |  |  | **B*56** | 2 (1.9) | / | / |  |  |  |  |
|  |  |  |  | **B*57** | 3 (2.8) | 2 (4) | 0.69 |  |  |  |  |
|  |  |  |  | **B*58** | 22 (20.8) | 10 (20) | 0.91 |  |  |  |  |
|  |  |  |  | **B*81** | / | 1 (2) | / |  |  |  |  |

*P-value from chi-square/fisher test. Statistically significant results are marked in bold. /: Not applicable. *N: number of phenotype*
