# Supplementary material for: HLA A*32 is associated to HIV acquisition while B*44 and B*53 are associated with protection against HIV acquisition in perinatally exposed infants
Source: BMC Pediatr. 2019 Jul 23;19:249. doi: 10.1186/s12887-019-1620-6 (PMC6647251; doi:10.1186/s12887-019-1620-6)
Supplement: Supplementary file 5 — : Table S5. HLA class 1 ABC distribution in the HIV exposed infected and exposed non-infected babies. (DOCX 34 kb) [file 12887_2019_1620_MOESM5_ESM.docx]

**Additional file 5: Table S5:** HLA class 1 ABC distribution in the HIV exposed infected and exposed non-infected babies

| **HLA class A** | **Phenotypic frequency**  **[N(%]** | | ***P** | **HLA class B** | **Phenotypic frequency**  **[N(%]** | | ***P** | **HLA class C** | **Phenotypic frequency**  **[N(%]** | | ***P** |
| --- | --- | --- | --- | --- | --- | --- | --- | --- | --- | --- | --- |
|  | **EI** | **ENI** |  |  | **EI** | **ENI** |  |  | **EI** | **ENI** |  |
| **A*01** | 8 (19.0) | 6 (9.4) | 0.08 | **B*07** | 10 (23.8) | 14 (21.9) | 0.18 | **C*01** | 1 (2.4) | 1 (1.6) | 0.48 |
| **A*02** | 17 (40.5) | 34 (53.1) | 0.07 | **B*08** | 2 (4.8) | 1 (1.6) | 0.28 | **C*02** | 8 (19.0) | 20 (31.3) | 0.06 |
| **A*03** | 5 (11.9) | 8 (12.5) | 0.23 | **B*13** | 1 (2.4) | 1 (1.6) | 0.48 | **C*03** | 5 (11.9) | 5 (7.8) | 0.20 |
| **A*11** | 1 (2.4) | 2(3.1) | 0.43 | **B*14** | 4 (9.5) | 1 (1.6) | 0.07 | **C*04** | 12 (28.6) | 20 (31.3) | 0.16 |
| **A*21** | 1 (2,4) | / | / | **B*15** | 5 (11.9) | 9 (14.1) | 0.22 | **C*05** | / | 3 (4.7) | / |
| **A*23** | 2 (4,8) | 6 (9.4) | 0.21 | **B*18** | 1 (2.4) | 5 (7.8) | 0.18 | **C*06** | 12 (28.6) | 17 (26.6) | 0.17 |
| **A*24** | 1 (2,4) | 4 (6.3) | 0.26 | **B*27** | 3 (7.1) | 3 (4.7) | 0.28 | **C*07** | 17 (40.5) | 28 (43.8) | 0.15 |
| **A*25** | 1 (2,4) | / | / | **B*35** | 11 (26.2) | 13 (20.3) | 0.14 | **C*08** | 4 (9.5) | 6 (9.4) | 0.26 |
| **A*26** | 2 (4,8) | 4 (6.3) | 0.32 | **B*37** | 2 (4.8) | 1 (1.6) | 0.28 | **C*12** | 3 (7.1) | 7 (10.9) | 0.22 |
| **A*28** | / | 2 (3.1) | / | **B*38** | 2 (4.8) | 1 (1.6) | 0.28 | **C*14** | 5 (11.9) | 5 (7.8) | 0.20 |
| **A*29** | 6 (14.3) | 8 (12.5) | 0.21 | **B*39** | / | 1 (1.6) | / | **C*15** | 1 (2.4) | 1 (1.6) | 0.48 |
| **A*30** | 9 (21.4) | 14 (21.9) | 0.18 | **B*40** | 2 (4.8) | 4 (6.3) | 0.32 | **C*16** | 3 (7.1) | 4 (6.3) | 0.29 |
| **A*31** | 4 (9.5) | 2 (3.1) | 0.13 | **B*41** | / | 1 (1.6) | / | **C*17** | 1 (2.4) | 6 (9.4) | 0.15 |
| **A*32** | **7 (16.7)** | **1 (1.6)** | **0.005** | **B*42** | 1 (2,4) | 4 (6.3) | 0.26 | **C*18** | 1 (2.4) | 2 (3.1) | 0.43 |
| **A*33** | 4 (9.5) | 2 (3.1) | 0.13 | **B*44** | **11 (26.2)** | **13 (42.2)** | **0.04** |  |  |  |  |
| **A*34** | 1 (2.4) | 2 (3.1) | 0.43 | **B*45** | 3 (7.1) | 3 (4.7) | 0.58 |  |  |  |  |
| **A*36** | 1 (2.4) | 7 (10.9) | 0.08 | **B*46** | 1 (2.4) | / | / |  |  |  |  |
| **A*66** | 3 (7.1) | 3 (4.7) | 0.28 | **B*47** | 3 (7.1) | 3 (4.7) | 0.28 |  |  |  |  |
| **A*68** | 3 (7.1) | 7 (10.9) | 0.22 | **B*48** | 1 (2.4) | 2 (3.1) | 0.43 |  |  |  |  |
| **A*74** | / | 4 (6.3) | / | **B*49** | 2 (4.8) | 5 (7.8) | 0.26 |  |  |  |  |
| **A*80** | 1 (2.4) | / | / | **B*50** | / | 2 (3.1) | / |  |  |  |  |
|  |  |  |  | **B*51** | 3 (7.1) | 5 (7.8) | 0.29 |  |  |  |  |
|  |  |  |  | **B*52** | 1 (2.4) | / | / |  |  |  |  |
|  |  |  |  | **B*53** | **1 (2.4)** | **9 (14.1)** | **0.03** |  |  |  |  |
|  |  |  |  | **B*56** | / | 2 (3.1) | / |  |  |  |  |
|  |  |  |  | **B*57** | 2 (4.8) | 1 (1.6) | 0.28 |  |  |  |  |
|  |  |  |  | **B*58** | 10 (23.8) | 12 (18.8) | 0.15 |  |  |  |  |

*P-value from chi-square/fisher test; ENI: Exposed non-infected; EI: Exposed infected; /: Not applicable. Statistically significant results are marked in bold.

*N: number of phenotype*
